# Mapping prohormone processing by proteases in human enteroendocrine cells using genetically engineered organoid models

**DOI:** 10.1073/pnas.2212057119

**Published:** 2022-11-07

**Authors:** Joep Beumer, Julia Bauzá-Martinez, Tim S. Veth, Veerle Geurts, Charelle Boot, Hannah Gilliam-Vigh, Steen S. Poulsen, Filip K. Knop, Wei Wu, Hans Clevers

**Affiliations:** ^a^Hubrecht Institute, Royal Netherlands Academy of Arts and Sciences (KNAW) and University Medical Center (UMC) Utrecht, Oncode Institute, 3584 CT Utrecht, The Netherlands;; ^b^Biomolecular Mass Spectrometry and Proteomics, Bijvoet Center for Biomolecular Research and Utrecht Institute for Pharmaceutical Sciences, Utrecht University, 3584 CH Utrecht, The Netherlands;; ^c^Center for Clinical Metabolic Research, Gentofte Hospital, University of Copenhagen, 2900 Hellerup, Denmark;; ^d^Department of Biomedical Sciences, Faculty of Health and Medical Sciences, University of Copenhagen, 2200 Copenhagen, Denmark;; ^e^Steno Diabetes Center Copenhagen, 2730 Herlev, Denmark;; ^f^Department of Clinical Medicine, Faculty of Health and Medical Sciences, University of Copenhagen, 2200 Copenhagen, Denmark;; ^g^Singapore Immunology Network (SIgN), Agency for Science, Technology and Research (A*STAR), Singapore 138648, Singapore;; ^h^Department of Pharmacy, National University of Singapore, Singapore 117543, Singapore

**Keywords:** enteroendocrine cells, intestinal organoids, CRISPR-Cas9, prohormone processing, peptidomics

## Abstract

Enteroendocrine cells (EECs) secrete hormones in response to ingested nutrients to control physiological processes such as appetite and insulin release. EEC hormones are synthesized as large proproteins that undergo proteolytic processing to generate bioactive peptides. Mutations in EEC-enriched proteases are associated with endocrinopathies. Due to the relative rarity of EECs and a paucity of in vitro models, intestinal prohormone processing remains challenging to assess. Here, human gut organoids in which EECs can efficiently be induced are subjected to CRISPR-Cas9–mediated modification of EEC-expressed endopeptidase and exopeptidase genes. We employ mass spectrometry–based analyses to monitor peptide processing and identify glucagon production in intestinal EECs, stimulated upon bone morphogenic protein (BMP) signaling. We map the substrates and products of major EECs endo- and exopeptidases. Our studies provide a comprehensive description of peptide hormones produced by human EECs and define the roles of specific proteases in their generation.

## Significance

Enteroendocrine cells control key physiological processes such as appetite and insulin secretion through the secretion of neurotransmitters and peptide hormones. The bioactive peptides are subject to complex proteolytic processing essential for their activation or inactivation. Moreover, alternative processing allows for the generation of different peptides from the same precursor protein. We use human organoid cultures combined with CRISPR-Cas9–mediated loss of function and peptidomics to assay the peptide spectrum of gut proteases. We identify substrates of these enzymes and identify the production of intestinal glucagon in organoids. A more complete understanding of hormone processing could allow a rational design of therapeutic interventions targeting these proteases.

The mammalian intestine constitutes the largest hormone-producing organ through the activity of enteroendocrine cells (EECs) (1). EECs represent only 1% of the intestinal epithelium. The >20 known bioactive products released from these cells regulate key physiological processes including appetite, insulin secretion, and bowel movement. EECs secrete their products in response to luminal or absorbed nutrients (2). The EEC products are believed to signal in a paracrine manner, locally to the enteric nervous system and musculature and to distant endocrine organs and the central nervous system.

EECs synthesize prohormone proteins from which bioactive peptides are derived through complex posttranslational processing by endopeptidases and exopeptidases (3). The majority of these proteases colocalize with hormones in the secretory pathway of the EECs. Multiple EEC-enriched proteases involved in hormone processing have been described. The calcium-regulated proprotein convertase PCSK1 (also known as PC1/3), a serine endoprotease, cleaves substrates C terminally to dibasic residues and is essential for the generation of the incretin glucagon-like peptide 1 (GLP-1) from preproglucagon (encoded by *GCG*) in intestinal L cells, a subtype of EECs (4). Pancreas islet-specific PCSK2 (PC2) provides alpha cells with the unique capacity to produce glucagon from the same prohormone. Carboxypeptidases cleave C-terminal basic residues generated after the endoprotease activity (5). EECs express the carboxypeptidase CPE. After the secretion of bioactive peptides, extracellular proteases can further process hormones to regulate their activity. Most notably, transmembrane dipeptidyl peptidase 4 (DPP4) has N-terminal exopeptidase activity and is best known for its ability to inactivate GLP-1 (6). DPP4 inhibitors, which are successful type 2 diabetes drugs, prevent inactivation of secreted GLP-1.

Mutations in EEC-enriched proteases are an important cause of endocrinopathies. For example, alterations in PCSK1 are linked to severe metabolic disorders (4, 7). No accurate models nor treatments have been developed for these diseases. While these proteases have expression profiles beyond the gut, mapping their intestinal substrate spectrum may have clinical implications for these inherited diseases. Vice versa, pharmacological activation or inhibition of such enzymes could hold therapeutic potential for a range of diseases including type 2 diabetes, similar to DPP4 inhibitors. The functions of EEC-enriched endopeptidases or exopeptidases have mostly been assessed using mouse models. For example, CPE mutant mice develop broad metabolic symptoms including diabetes. Yet, EEC-specific processing defects were not studied in this model (8). Since human EECs produce several unique hormones compared to mouse EECs (e.g., motilin [MLN] and neuropeptide W [NPW]), mouse models have additional shortcomings to fully map the substrate specificity in human EECs. Moreover, we have previously identified a human EEC-enriched carboxypeptidase, CPB1, that is not produced by mice (9). An alternative approach has involved coexpression of a wildtype or mutant forms of proteases with the prohormones in cell lines (10). Although such models allow more experimental flexibility, results might not reflect the endogenous processing occurring in the secretory pathway of human EECs.

We have previously generated a toolset for the study of human EECs in adult stem cell–derived organoid cultures (9). The conditional overexpression of the proendocrine transcription factor Neurogenin-3 allows for the generation of high-purity cultures of EECs, enabling their functional study. This system allows for an in-depth study of human EECs, which includes the analysis of their secreted products. Here, we employ CRISPR-Cas9–mediated gene editing, efficient EEC differentiation, and mass spectrometry–based peptidomic analysis to determine the role of endogenous gut proteases in hormone processing.

## Results

**Fig. 1.**
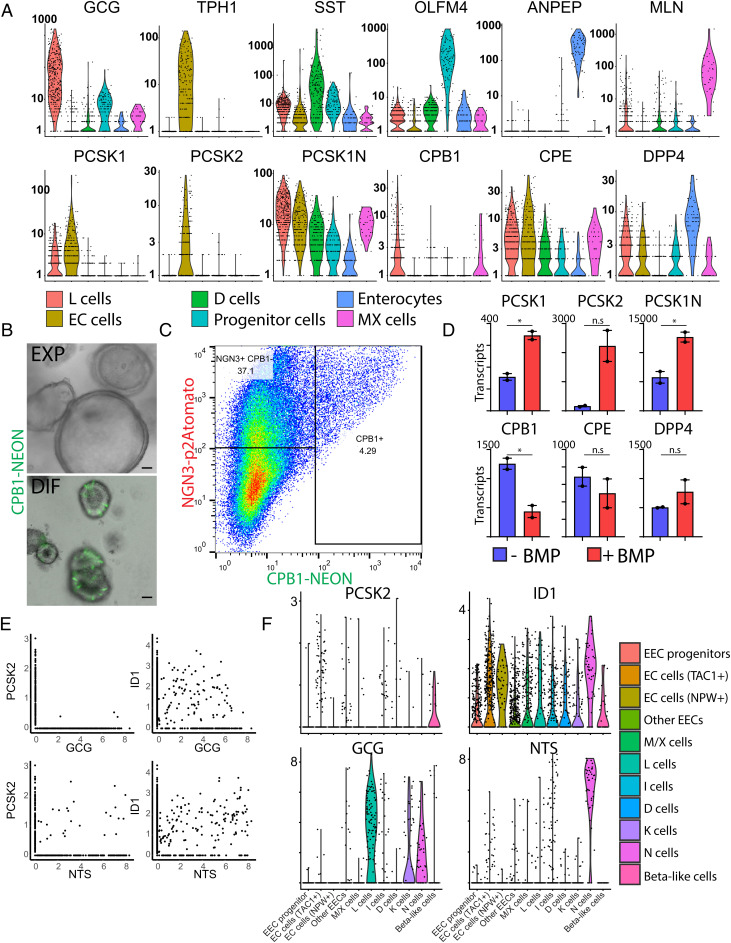
Expression profile of EEC-enriched proteases. (*A*) Violin plots depicting protease gene expression levels in different EEC populations derived from organoid cultures based on previously performed single-cell RNA-sequencing (9). (*B*) Overlay of brightfield and fluorescent image from CPB1^mNeon^ organoids in culture. CPB1-neon positive cells are only observed upon EEC differentiation (DIF) and not in expanding conditions (EXP). Scale bars, 50 μm. (*C*) Fluorescence-activated cell sorting analysis of differentiated NEUROG3^tdTomato/^CPB1^mNeon^ organoids. Neon-positive cells can only be detected in NEUROG3-overexpressing cells, suggesting restricted expression to EECs. (*D*) Graphs depicting protease expression revealed by bulk RNA-sequencing of EEC-differentiated organoids in the presence and absence of BMPs. (*E*) Hormone and protease expression plotted from a previously published single-cell RNA-sequencing atlas of the gut epithelium (21). (*F*) Violin plots depicting *PCSK2*, BMP target *ID1*, and the hormones *GCG* and *NTS* expression levels in different EEC populations from a previously published single-cell RNA-sequencing atlas of the gut epithelium (21).

We first assessed protease expression in different human EEC subtypes in ileal-derived organoids by using our previously generated single-cell RNA-sequencing dataset (9). The highest PCSK1 expression was observed in GLP-1-producing L cells as well as in enterochromaffin cells (ECs), which secrete the neurotransmitter 5-HT (serotonin). PCSK2 was mostly restricted to ECs (Fig. 1*A* and *SI Appendix*, Fig. S1 *A* and *B*). The *PCSK1N* gene codes for multiple bioactive peptides including proSAAS, which negatively regulates PCSK1 activity and is broadly expressed by all EEC populations (11). The transmembrane and soluble protease DPP4 is not restricted to EECs but is produced by different intestinal lineages, most strongly by enterocytes (Fig. 1*A* and *SI Appendix*, Fig. S1 *A* and *B*). We observed the expression of the carboxypeptidase CPE in all EEC populations. A second carboxypeptidase, CPB1, was most prominently expressed in L cells and MLN/ghrelin-producing MX cells. CPB1 is not expressed in murine EECs, and its function is not known (Fig. 1*A* and *SI Appendix*, Fig. S1 *A* and *B*). To confirm its EEC-specific expression profile, we generated a knock-in reporter by targeting CRISPR-Cas9 to the CPB1 stop codon followed by nonhomologous end joining introducing fluorescent mNeon (12). CPB1^mNeon+^ cells were rarely observed in organoids in expansion conditions when EECs are mostly absent. When organoids were differentiated to form EECs, we observed the expression of CPB1 in a subset of EECs (Fig. 1 *B* and *C*). We generated an identical reporter construct for the known EEC protease PCSK1 that displayed a similar pattern (*SI Appendix*, Fig. S1*C*).

Some of the EEC subtypes alter the expression of hormones while traveling from crypt, where they are born, to villus tips (13). L cells express the *GCG* gene in crypts but switch to a cell type that enriches for neurotensin (*NTS*) when migrating toward the tip of the villus. This phenotype switch is controlled by a bone morphogenic protein (BMP) signaling gradient, which is inactive at crypt bottoms and highly active at villus tips (13, 14). Bulk RNA-sequencing was performed on EEC-differentiated organoids in the presence and absence of BMP (*SI Appendix*, Datasets S1 and S2). As previously reported (13, 15), we observed strong down-regulation by BMP signals of crypt hormone *GCG* in L cells, while villus hormones *NTS* and *SCT* increased (*SI Appendix*, Fig. S1*D*). Additionally, the recently discovered EEC product NPW was strongly induced by BMP activation (*SI Appendix*, Fig. S1*D*) (9, 16). Accordingly, we found that EECs from native human gut tissue producing the highest level of *NPW* displayed expression of the BMP target gene *ID1* (*SI Appendix*, Fig. S1*E*). NPW is expressed in ECs. The total numbers of ECs were not affected by BMP, as based on *TPH1* expression, which is the rate-limiting enzyme for 5-HT production (*SI Appendix*, Fig. S1*D*). The majority of proteases did not display strong differential expression upon BMP activation, with the exception of BMP-induced *PCSK2* (Fig. 1*D*). PCSK2 is known to catalyze the production of glucagon in the pancreas (17). PCSK2 has been suggested to process GIP in murine intestinal K-cells (18) and has been identified in murine ECs and—albeit lowly—in canine L cells (19, 20). We analyzed a recently published single-cell sequencing atlas of the human gastrointestinal tract to assess *PCSK2* expression in the different EEC populations (21). The strongest expression was observed in adult ECs, as expected, and in fetal EECs producing insulin (Fig. 1 *E* and *F*). In addition, we detected rare coexpression of *PCSK2* in L cells producing *GCG*, and higher levels in *NTS*^+^ EECs (Fig. 1 *E* and *F* and *SI Appendix*, Fig. S1*F*). L cells produce small amounts of *NTS* in the crypt but increase its expression while experiencing higher levels of BMP (13, 14). *NTS*^+^ cells indeed expressed the BMP target gene *ID1* (Fig. 1 *E* and *F*). We sorted *NTS*^+^ cells by using a previously established reporter organoid reporter line and found that BMP activation within these cells stimulated *PCSK2* (*SI Appendix*, Fig. S1*G*). These findings raised the possibility that crypt L cells may produce small amounts of PCSK2 and thus of glucagon (in addition to the prototypic L cell product GLP-1) in the crypt. Villus L cells down-regulate *GCG* expression and yet could have an increased capacity to process pre-existing proglucagon to glucagon because of increasing PCSK2 expression.

**Fig. 2.**
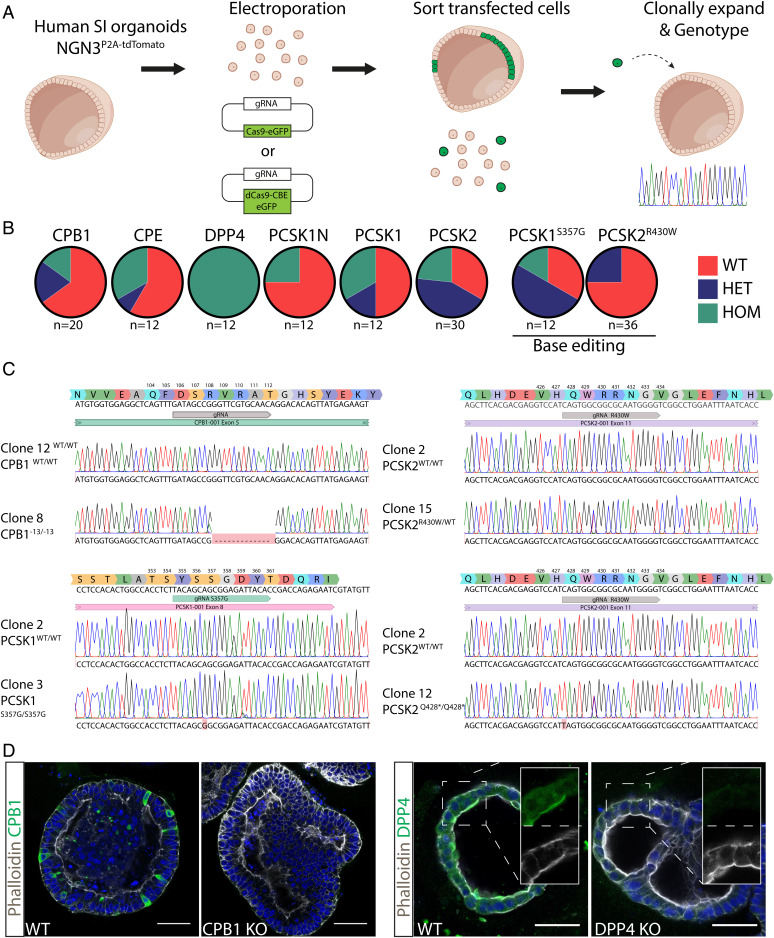
Genetically engineered organoids harboring protease mutations. (*A*) Cartoon displaying workflow to generate protease mutant organoids. (*B*) Targeting efficiency of the different genotypes. In-frame mutations were not counted as targeted due to the probable lack of phenotype. (*C*) Sanger traces of protease mutant clones. (*D*) Immunofluorescent staining of CPB1 and DPP4 mutant organoids. Stainings confirm genotype and absence of the targeted proteins. Scale bars, 50 μm.

We employed CRISPR-Cas9–mediated gene editing to generate loss-of-function mutants of the various EEC proteases (*SI Appendix,* Table S1). We included the endopeptidases PCSK1 and PCSK2; the carboxypeptidases CPE and CPB1; the aminopeptidase DPP4; and finally PCSK1N, a regulator of PCSK1 activity and potentially of other proteases (Fig. 2*A*). In addition, we used cytosine base editors to introduce previously reported point mutations in proteases as they occur in hereditary disorders. We included mutations reported to alter specificity rather than loss-of-function defects. Thus, we generated targeting constructs to produce the PCSK1^S357G^ mutation, which is suggested to be a hypermorph mutant with a PCSK2-like substrate spectrum (10). Secondly, we generated PCSK2^R430W^ mutations, which were previously suggested to cause increased conversion to type 2 diabetes in Amish subjects (22). We transfected organoid cells with CRISPR-Cas9 plus a protease-specific guide RNA (gRNA) to induce frameshifts or with CRISPR-Cas9 base editor combinations (Fig. 2*A*). Transfected cells were sorted based on GFP expression, clonally expanded, and genotyped for the appropriate genotype (Fig. 2*A*). Clonal knockout organoid lines were generated for all proteases and point mutations. For PCSK2^R430W^, we only recovered a heterozygous line (Fig. 2 *B* and *C*). Nonetheless, we proceeded with this organoid line as the phenotype is predicted to be a gain of function. We also generated PCSK1/PCSK2 double-mutant organoids by sequential transfection to assess potential redundancy between these proteases. We confirmed a complete loss of protein production for the CPB1 and DPP4 knockouts (Fig. 2*D*).

We next employed mass spectrometry to document the proteolytic processing of gut hormones. We first focused our analyses on mutants of the endopeptidases. Wild-type and mutant organoid lines were differentiated in the presence and absence of BMPs, allowing the generation of the complete spectrum of EEC states and hormones as observed in vivo (*SI Appendix*, Fig. S2*A*). We did not note major morphological differences between wild-type and mutant organoids, with the exception of PCSK1/PCSK2 double-mutant organoids; while all organoids appeared cystic under expansion conditions, PCSK1^−/−^/PCSK2^−/−^ organoids displayed a dense phenotype with extensive budding (*SI Appendix*, Fig. S2*B*). Additionally, we always derived homozygous frameshift DPP4 mutants in this and a previous study, suggesting improved clonal outgrowth (Fig. 2*B*). We did not note altered splitting ratios in these mutants. These observations suggest that (unknown) substrates of DPP4, PCSK1, and PCSK2 could play a role in clonal outgrowth, cell proliferation, or morphogenetic events.

**Fig. 3.**
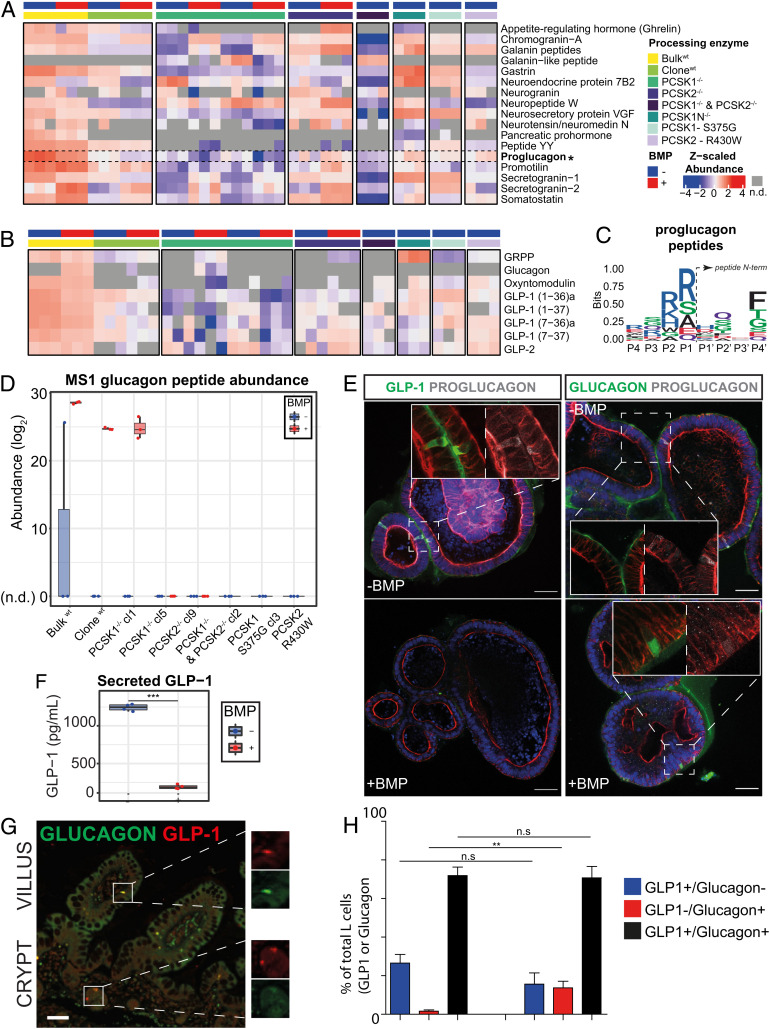
BMP-induced glucagon production in human EECs. (*A*) LC-MS/MS abundance profiles of the diverse prohormones measured in the intracellular peptidome of wild-type and mutant organoids. Concentration of measured peptides was higher in the parental, nonclonal organoid (bulk) compared to clonal lines due to higher differentiation efficiency. Abundance is Z-scaled. Samples where the prohormone was not detected (n.d.) are displayed in gray. BMP-untreated (−) is shown in blue and BMP-treated (+) is shown in red. (*B*) LC-MS/MS abundance profiles of the bioactive peptides derived from proglucagon. For amidated peptides, an “a” is annotated after the peptide name. Abundance is Z-scaled. Samples where the prohormone was not detected (n.d.) are displayed in gray. BMP-untreated (−) is shown in blue and BMP-treated (+) is shown in red. (*C*) Sequence logo of residues found around the N termini of all peptides derived from proglucagon. Dibasic residues are enriched at cleavage site (P1, P2) just before the N termini (P1’, P2’, P3′, P4’) of peptides mapping to proglucagon, reflecting PCSK1 and PCSK2 cleavage specificities. Peptides measured in wild-type organoids were used, and any duplicate N-termini sequences (P-4 to P4) were excluded. In total, 11 unique N termini (P-4 to P4) were used to generate the logo plot. (*D*) Total abundance for the glucagon peptide as extracted from the MS1 isotopic distributions evaluated on Skyline. Total abundance was calculated by summation of the individual intensities of all isotopes. Total abundance is displayed in log2 space. Each point in the boxplot represents individual injection replicates. BMP-untreated (−) is shown in blue and BMP-treated (+) is shown in red. When no reliable isotopic distribution was detected (n.d.), abundance was set to zero. Individual isotopic distributions for glucagon are shown in *SI Appendix*, Fig. S3, and the Skyline result file has been deposited in the PRIDE Repository. (*E*) Immunofluorescent staining of proglucagon, GLP-1, and glucagon in BMP-untreated and -treated organoids. Scale bars, 50 μm. (*F*) GLP-1 ELISA on the supernatant of organoids differentiated in the presence and absence of BMPs. Organoids were stimulated to secrete for 6 h using forskolin and IBMX. Statistical significance was determined by two-sided Student’s *t* test (***P < 0.001). (*G*) Immunofluorescent staining of proglucagon and GLP-1 on human intestine. Scale bars, 50 μm. (*H*) Quantification of *G*.

Next, we collected the intracellular peptidome from differentiated organoids of all mutants. We additionally collected secreted peptides from all organoid models, which were induced to secrete their hormones for 90 min by chemical stimulation with adenylyl cyclase, inducing intracellular cAMP levels. We included both the parental and a wild-type sibling organoid line for comparison. Due to the higher quality data extracted from intracellular peptidomes, which are naturally protected from unspecific proteolytic events happening extracellularly upon secretion, we focused our analyses on this fraction. The introduced mutations as well as BMP status caused major shifts in the observed number of processed bioactive peptides (Fig. 3*A*). PCSK1^−/−^/PCSK2^−/−^ peptidomes displayed a reduction in the most bioactive peptides, which were stronger than single PCSK1 or PCSK2 mutants (Fig. 3*A*). This indicated functional redundancy between these proteases. PCSK1 single mutants displayed a reduction in multiple peptides mapping to gastrin, promotilin (a hormone existing as a pseudogene in the mouse genome), and proglucagon. All of them were qualitatively increased in PCSK1N mutants, coding for a known negative regulator of PCSK1 activity (Fig. 3*A*) (11). We did not observe major changes in peptides extracted from PCSK1^S357G^ and PCSK2^R430W^ organoids, suggesting a minimal impact on enzymatic activity of these reported point mutations. Proglucagon is known to be heavily modified by the endopeptidases PCSK1 and PCSK2 (23), which cleave prohormones after dibasic residues (lysine [K] and arginine [R]). This is reflected in the strong enrichment of dibasic residues just before the N termini of peptides mapping to proglucagon (Fig. 3*C*, positions P1 and P2). Focusing on bioactive peptides derived from proglucagon, we noted a reduction of the different peptides in PCSK1 null mutants (Fig. 3*B*). BMP activation negatively impacted the total levels of proglucagon (Fig. 3 *A* and *B*), reflecting its effect on *GCG* transcription. Surprisingly, we detected bioactive glucagon specifically in BMP-treated conditions (Fig. 3*B* and *SI Appendix*, Fig. S3), which is in line with the induction of *PCSK2* transcription by BMP (Fig. 1*D*). We did not observe significant amounts of glucagon in the majority of BMP-inhibited cultures nor in any of the BMP-activated PCSK2 mutant organoids (Fig. 3*D*). Glucagon was only absent in one of the two PCSK1 mutant lines, potentially hinting to a role of this enzyme in its production. In addition to spectral evidence of glucagon obtained with our peptidomics approach, we further corroborated this evidence with MS1 and MS2 traces obtained using a parallel reaction monitoring (PRM) mass spectrometry approach at optimal fragmentation and resolution (*SI Appendix*, Figs. S4 and S5). This approach yielded near-complete fragmentation of the glucagon peptide (*SI Appendix*, Fig. S4) and demonstrated coeluting MS1 and MS2 traces found in the wild-type, EEC-differentiated organoids for both glucagon and active GLP-1 (7-37 and 7-36a) (*SI Appendix*, Fig. S5*A*). This confirmed the BMP-dependent increase of glucagon production and reduction in GLP-1 production (*SI Appendix*, Fig. S5*B*).

To validate mass spectrometry findings, we performed immunofluorescence by using glucagon- and GLP-1-specific antibodies, as well as an antibody recognizing unprocessed proglucagon. We found that virtually all proglucagon-positive cells in BMP-inactive conditions showed GLP-1 (but not glucagon) colocalization (Fig. 3*E*). By contrast, BMP activation drastically reduced the number of proglucagon cells and intensity, but the remaining positive cells displayed glucagon reactivity. An analysis of secreted peptides by enzyme-linked immunosorbent assay (ELISA) confirmed the BMP-mediated reduction of GLP-1 levels. Secreted glucagon remained below the detection threshold (Fig. 3*F*). To assess the potential BMP regulation of proglucagon-derived peptides in human tissue, we stained intestinal sections for Glucagon and GLP-1. The fraction of L cells that only produced Glucagon and not GLP-1 changed strongly from crypt to villus (Fig. 3 *G* and *H*). We conclude that human EECs are capable of producing bioactive glucagon, potentially mediated by BMP-stimulated expression of PCSK2.

**Fig. 4.**
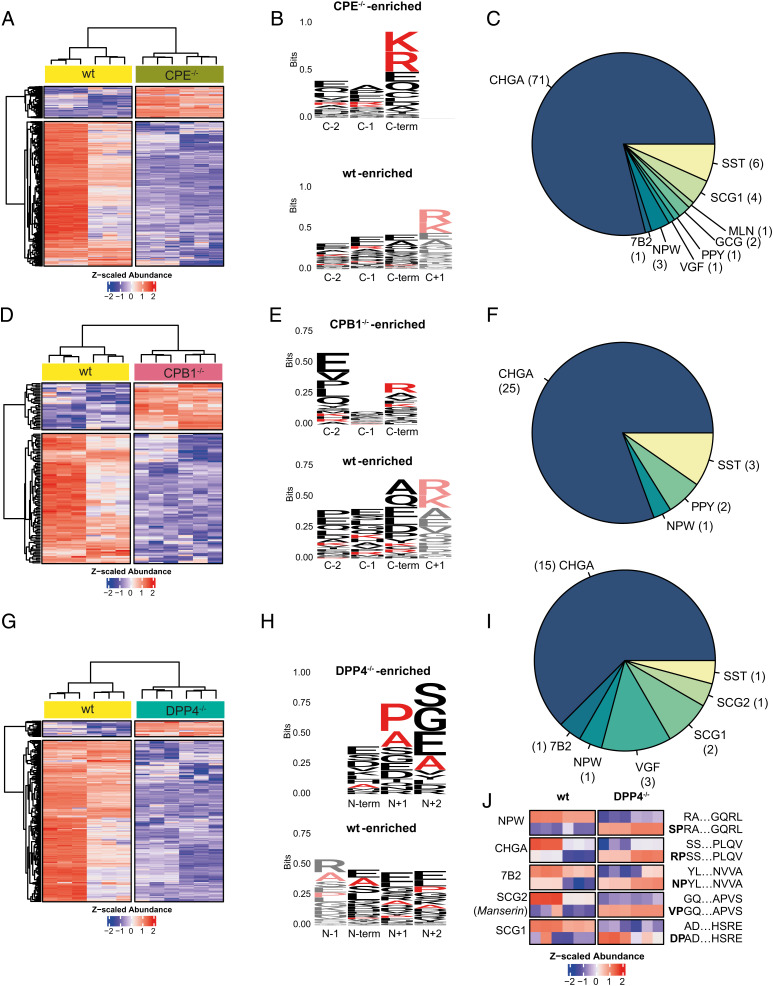
Intracellular substrate specificity of EEC exopeptidases. (*A*) Significantly changing hormone-derived peptides in response to CPE knockout (CPE^−/−^) when compared to wild-type organoids. Bulk and a clonal wildtype line and two CPE^−/−^ clones were included. Three technical replicates were measured for all samples. (*B*) Sequence logo of the C termini of peptides accumulating in response to CPE knockout (CPE^−/−^-enriched) and peptides accumulating in the wild type (wt-enriched). K and R are highlighted in red. (*C*) Pie chart indicating the substrate repertoire of peptides accumulating in CPE knockouts. (*D*) Significantly changing hormone-derived peptides in response to CPB1 knockout (CPB1^−/−^) when compared to wild-type organoids. Bulk and a clonal wild-type line and two CPB1^−/−^ clones were included. Three technical replicates were measured for all samples. (*E*) Sequence logo of the C termini of peptides accumulating in response to CPB knockout (CPB^−/−^-enriched) and peptides accumulating in the wild type (wt-enriched). K and R are highlighted in red. (*F*) Pie chart indicating the substrate repertoire of peptides accumulating in CPB1 knockouts. (*G*) Significantly changing hormone-derived peptides in response to DPP4 knockout (DPP4^−/−^) when compared to wild-type organoids. Bulk and a clonal wild-type line and two DPP4^−/−^ clones were included. Three technical replicates were measured for all samples. (*H*) Sequence logo of the N-termini of peptides accumulating in response to DPP4 knockout (DPP4^−/−^-enriched) and peptides accumulating in the wild type (wt-enriched). P and A are highlighted in red. (*I*) Pie chart indicating the substrate repertoire of peptides accumulating in DPP4 knockouts. (*J*) Heatmap showing the Z-scaled abundance of peptide pairs differing by XP/A dipeptide cleavage when comparing DPP4^−/−^ to wild-type clones.

Next, we analyzed the substrate spectrum of the carboxypeptidases CPE and CPB1. Loss of CPE resulted in the accumulation of 90 peptides mapping to multiple prohormones (Fig. 4*A*). These peptides were strongly enriched in the basic residues K and R at the C-terminal position (Fig. 4*B*). Notably, the set of peptides enriched in wild-type organoids compared to CPE mutants did not present K or R in the C-terminal position but did in the contiguous residue (C-terminal +1) (Fig. 4*B*). Among the peptides accumulating in the CPE^−/−^ background, we found sequences derived from NPW (a hormone not observed in the murine gut), VGF, the PC2 chaperone neuroendocrine protein 72B, proglucagon, promotilin, pancreatic prohormone (PPY), somatostatin (SST), and chromogranin A (Fig. 4*C*). In the secretomes of wild-type and CPE mutant organoids, we observed a slightly larger range of substrates (*SI Appendix*, Fig. S6 *A–C*), indicating that this carboxypeptidase acts both intra- and extracellularly.

Deletion of the human EEC-specific CPB1 resulted in the accumulation of a smaller set of 31 hormone-derived peptides (Fig. 4*D*), consistent with a narrower expression pattern as well as lower levels of this carboxypeptidase compared to CPE (Fig. 1*A*). Accumulating peptides displayed C-terminal enrichment of the basic amino acids R and K (Fig. 4*E*), although other residues also occurred in this position. Notably, we observed an accumulation of SST, NPW, and PPY peptides upon loss of CPB1 (Fig. 4*F*). PPY-derived peptides included the C-terminal amidated versions of the full and partial sequence of the pancreatic hormone bioactive region (residues 30 to 65 and 37 to 51; *SI Appendix*, Dataset S3). Another bioactive region of PPY, termed pancreatic icosapeptide, was similarly enriched in one of the knockout clones of CPB1 when compared to the wild type. We measured the extracellular peptidome secreted by CPB1^−/−^ organoids but could not find significantly enriched sequences. Although this is potentially due to sensitivity and the low levels of extracellular peptides, this observation may also result from low extracellular activity of this carboxypeptidase.

We next investigated the peptide spectrum related to the DPP4 enzyme. Unlike the carboxypeptidases, DPP4 is a membrane-anchored and secreted aminopeptidase that cleaves dipeptides from the N terminus of longer peptide substrates (24). As a transmembrane protein, DPP4 is believed to mainly act on extracellular substrates, including secreted peptide hormones such as GLP-1 (25). We surprisingly find intracellular accumulation of multiple hormone-derived peptides in DPP4 mutants (Fig. 4 *G*–*J*). Validating these observations, we found a strong enrichment of proline (P) and alanine (A) residues directly adjacent to the N-terminal residue (Fig. 4*H*) in accumulated peptides. The enrichment of P/A in position N+1 is in line with the reported cleavage site specificity for DPP4 (26). We found a broad range of intracellular substrates for DPP4 (Fig. 4*I* and *SI Appendix*, Dataset S3). Among these, we identified peptide pairs that only differed by the cleavage of the XP/A dipeptide when comparing DPP4^−/−^ to wild-type clones (Fig. 4*J*). These peptides indicated intracellular DPP4 activity, as the cleaved versions were enriched in the wild type, whereas the XP/A-containing versions were enriched in the DPP4 knockouts. For example, one of the few bioactive products of secretogranin-2 (SCG2), named manserin, was found as a direct substrate of DPP4 (Fig. 4*J*). We identified extracellular targets of DPP4 covering a broader range of substrates and displaying lower cleavage specificity (*SI Appendix*, Fig. S6 *D–F*), which was reflected by lower enrichment of P/A in position N+1 of accumulating peptides (*SI Appendix*, Fig. S6*E*). This may result from nonspecific degradation of the secreted peptide hormones by additional extracellular (amino)peptidases.

## Discussion

Processing of bioactive peptides from large prohormones is a complex process involving a wide range of peptidases. Due to the obligatory colocalization of hormone and protease, as well as the expression of codependent enzymes, processing is ideally studied in a physiological setting. As hormone and protease production in mice and humans differ (9, 16), organoids provide the unique opportunity to assess prohormone processing in near-native human gut cells. By exploiting EEC enrichment in human organoids with CRISPR-Cas9 engineering and applying mass spectrometry–based peptidomic analysis on endogenous peptide products, we describe the substrate spectrum of known peptidases, as well as of the newly described carboxypeptidase CPB1 (*SI Appendix*, Fig. S8*A*). We have previously shown that the expression of hormone transcripts including *GCG*, *SCT*, and *NTS* (here, extended to *NPW*) is under the control of BMP signaling, and we now extend this observation to posttranslational proteolytic processing. Of note, we could not detect significant production of Secretin peptides, which is potentially related to the relatively low expression of the prohormone in ileal organoids compared to other hormones (*SI Appendix*, Fig. S1*D*). *SCT* additionally has a lower expression in human compared to mouse tissue (16). PCSK2 is strongly induced by BMP activation and accordingly enriched in villus EEC cells positive for NTS in vivo. Consequently, full-length glucagon was only observed under BMP-activated conditions in organoids. These findings support previous indications based on antibody staining (27) that the intestine—in addition to alpha cells in the pancreas—secretes bioactive glucagon. We now append this finding with convincing spectral evidence of processed bioactive peptides.

Previous liquid chromatography–mass spectrometry (LC-MS) analyses of native human intestinal mucosa could not identify pancreatic-like glucagon peptides. This could potentially be related to the relative scarcity of EECs complicating rare peptide detection, as the same study could not detect other abundant hormones in some tissue samples (such as duodenal Gastrin and stomach Ghrelin in different specimens) (16). Moreover, measurements from native tissue could contain peptides derived beyond the intestinal epithelium. Our data imply that EECs migrating from crypt to villus alter bioactive output from prohormones through dynamic proteolytic processing, in addition to shifts in hormone gene transcription (*SI Appendix*, Fig. S8*B*). This process could potentially be therapeutically targeted by using small-molecule BMP inhibitors. In addition to increasing proglucagon output per cell, PCSK1-dependent production of GLP-1 would be enhanced in comparison to glucagon. This could be beneficial for multiple metabolic diseases. In addition, our study unveils many peptides that are specifically processed by proteases without a known function. These datasets can act as a starting point to investigate potentially novel EEC-derived bioactive peptides. Taken together, our study describes the substrates of the EEC proteases and provides a framework for understanding the intestinal component of hormone-processing defects in endocrinopathies as well as developing therapeutics influencing this process.

Our study has several limitations. First, dynamics in peptide processing among EECs cannot be assessed in vivo for nonabundant hormones due to low sensitivity of peptidomics, hampering validation. We have previously shown that organoid EECs resemble their tissue counterparts, and in this study, we validate the expression profiles of EEC enzymes (Fig. 1). Notably, *PCSK2* expression is found in tissue EECs producing high amounts of *NTS*, which was previously shown to be villus counterparts of L cells (13–15). We accordingly only find PCSK2 expression in sorted BMP-activated *NTS-*producing cells. Moreover, although organoid cultures can be enriched for hormone-producing EECs, we are still working on detection limits for some of the bioactive peptides (including glucagon). We detected glucagon in one PCSK1 mutant line and not in PCSK2 mutants. We can thus not exclude a role of PCSK1 in glucagon generation in EECs. Irrespective of the protease involved, BMP was required for glucagon generation in all organoid lines assessed. Moreover, differentiation efficiency was variable between organoid clones; this was most apparent for the parental line where we consistently measured more hormone peptides. Although we do not know if this is caused by certain epigenetic features, we can compare the relative abundance of peptides within clones, and therefore, these types of analyses are not confounded. Finally, we limited our studies to the distal human small intestine and a single patient line. We opted for this region due to a broad representation of EEC subtypes, but future work could exploit organoid models to investigate region-specific processing along the gastrointestinal tract.

## Materials and Methods

### Cell Culture of Human Intestinal Organoids.

Tissue from the human ileum of a 74-y old male was obtained from the University Medical Center (UMC) Utrecht with informed consent of the patient. The patient was diagnosed with colon adenocarcinoma that was resected. A sample from nontransformed, normal mucosa was taken for this study. The study was approved by the UMC Utrecht (Utrecht, the Netherlands) ethical committee and was in accordance with the Declaration of Helsinki.

Human small intestinal cells were cultured as described previously (13, 28). Organoids harboring inducible Neurogenin-3 expression were differentiated toward EECs as described before, for 5 d in differentiation medium. For the first two days of differentiation, Neurogenin3-tdTomato expression was induced using doxycycline (1 μg/mL). BMP activation was achieved by withdrawing Noggin from the differentiation medium and the addition of BMP-2 (Peprotech, 50 ng/mL) and BMP-4 (Peprotech, 50ng/mL). BMP ligands were added throughout the 5-d differentiation protocol. Medium was replaced every second day during the entire differentiation.

For the preparation of EEC secretomes, organoids were first washed for 1 h in warm phosphate-buffered saline (PBS) containing 0.001% bovine serum albumin (BSA) and 10 mM D-Glucose. Organoids were next stimulated to secrete for 90 min in PBS supplemented with 0.001% BSA, 10 mM D-Glucose, 10 μM forskolin (Tocris), and 10 μM 3-isobutyl-1-methylxanthine (IBMX; Sigma). Medium containing secreted medium was collected and spun at 2,000 g for 5 min, after which the supernatant was snap frozen.

### Immunostaining.

Organoid staining was performed as described previously (13). Intact organoids were collected by gently dissolving the BME in ice-cold PBS. Material was subsequently fixed overnight at 4 °C in 4% paraformaldehyde (Sigma). Permeabilization and blocking were next performed by incubating organoids in PBS containing 0.5% Triton X-100 (Sigma) and 2% normal donkey serum (Jackson ImunoResearch) for 30 min at room temperature (RT). All stainings were performed in blocking buffer (2% normal donkey serum in PBS). For immunofluorescence, we used the following antibodies: mouse anti-CPB1 (1:100; R&D systems, MAB2897), goat anti-DPP4 (1:200; R&D systems, AF1180), mouse anti-GLP1 (1:100; Santa Cruz, sc-73508), rabbit anti-proglucagon (1;100; Cell signaling, CST8233), and mouse anti-glucagon (1:100; Mercodia, 50-5051). For immunofluorescence, organoids were incubated with the corresponding secondary antibodies Alexa 488-, 568-, and 647-conjugated anti-rabbit or anti-mouse (1:1,000; Thermo Fisher Scientific) and Phalloidin-Alexa-568 (A12380) (Thermo Fisher Scientific) in blocking buffer containing DAPI (1; 1,000, Invitrogen). Sections were embedded in Vectashield (Vector Labs) and imaged using an Sp8 confocal microscope (Leica). Image analysis was performed using ImageJ software. Image contrast was adjusted for visualization purposes.

### FISH Using RNAscope.

The expression profile of *PCSK2*, *NTS*, and *GCG* was visualized using the RNAScope Multiplex Fluorescent Reagent Kit v2 (Advanced Cell Diagnostics) according to the manufacturer’s protocols (29). In brief, paraffin-embedded sections of the human intestine were deparaffinized, treated with hydrogen peroxide for 10 min, and boiled in target retrieval buffer for 15 min before a 30-min protease treatment (30). Using probes for *PCSK2*, *NTS*, and *GCG*, expression was assessed. After amplification and detection using opal dyes, slides were counterstained with DAPI for 30 s, mounted using ProLong Gold Antifade Mountant (Thermo Fisher scientific), and imaged using a SP8 confocal fluorescent microscope (Leica).

### RNA-Sequencing and Analysis.

For data in Fig. 1*A* and *SI Appendix*, Fig. S1, we visualized the expression of selected markers in our previously published organoid EEC single-cell RNA-sequencing dataset (GSE146799). Cells with at least 500 uniquely expressed genes and fewer than 30% mitochondrial reads were included in the analysis. Data were clustered and visualized using Seurat (dims = 10).

For data in Fig. 1 *E* and *F*, we visualized the expression of selected markers from a previously published gut epithelium cell atlas by using identical cell type annotations.

For newly generated bulk RNA-sequencing, two independent samples of organoids were differentiated for 5 d toward EECs in the presence and absence of BMPs. All material was collected in Eppendorf tubes containing 350 μL RLT buffer (RNeasy kit, QIAGEN). RNA was extracted using the RNeasy mini kit (QIAGEN) following the manufacturer’s instructions. Sequencing libraries were generated using a modified CELseq2 protocol (31). Next, 75-bp paired-end sequencing of libraries was performed on an Illumina NextSeq platform.

For this newly generated bulk RNA-sequencing dataset, reads were mapped to the human GRCh37 genome assembly. The counted reads were filtered to exclude reads with identical library and molecule barcodes. Differential gene expression analysis was performed using the DESeq2 package (32).

### Genetically Engineered Organoid Models.

For inducing frameshift mutations, we designed gRNAs targeting the different proteases and cloned them into a SpCas9-EGFP vector (Addgene plasmid #48138) by using a protocol described before (33). Single guide RNAs (sgRNAs) were selected using the ATUM gRNA design tool (https://www.atum.bio/eCommerce/cas9/input) (*SI Appendix,* Table S2). For inducing point mutations, gRNAs were designed to generate C-to-T mutations within the editing window of cytosine base editors (*SI Appendix,* Table S2). These gRNAs were cloned into pSPgRNA using the same protocol (Addgene plasmid #47108). For generation of PCSK1 and CPB1 reporter organoids, gRNAs were directed close to the C terminus and cloned into pSPgRNA (Addgene plasmid #47108) (*SI Appendix,* Table S2).

To generate homozygous frameshift mutations in the different proteases, organoids were transfected with 15 μg SpCas9-EGFP plasmid containing the locus-specific sgRNA. To generate C-to-T base edits, organoids were transfected with 5 μg of the gRNA plasmid and 10 μg plasmid encoding the cytosine base editor and eGFP (Addgene plasmid #112100). To generate PCSK1 and CPB1 fluorescent reporter organoids, we followed a protocol described before (12). In brief, organoids were transfected with 5 μg of the locus-specific gRNA plasmid; 5 μg of a targeting plasmid encoding mNeon; and 5 μg of a frameselector encoding mCherry, Cas9, and a second gRNA linearizing the targeting plasmid.

Transient transfection using an NEPA21 electroporator was performed as described before (34). At 3 to 7 d after transfection, organoids were dissociated using TryplE (TryplE Express; Life Technologies) and sorted on a FACS-ARIA (BD Biosciences) instrument for GFP or mCherry positivity. After the sorting step, Rho kinase inhibitor (Y-27632 dihydrochloride; 10 μM, Abmole) was added for 1 wk to support single-cell outgrowth.

To establish protease mutant organoid lines, 12 to 48 organoids were picked 1 to 2 wk after sorting. Manually picked organoids were dissociated using TryplE and plated in ∼50 μL BME. Approximately 5 μL of material was left for genotyping. DNA was extracted from the cells using the Zymogen Quick-DNA microprep kit according to protocol. Regions around the sgRNA target site were amplified using Q5 high-fidelity polymerase (New England BioLabs) according to the manufacturer’s protocol. CRISPR-Cas9–mediated indel formation was confirmed by Sanger sequencing of these amplicons (Macrogen). Subsequently, Sanger trace deconvolution was performed with the use of ICE v2 CRISPR analysis tool to call clonal organoid lines with homozygous frameshift mutations at the target site. Heterozygous or homozygous base edits could be called based on immediate manual inspection of sequence. Primers used for amplification and Sanger sequencing can be found in *SI Appendix,* Table S2.

To establish stable reporter organoid lines, organoids displaying fluorescent cells were picked 1 to 2 wk after sorting. Although EECs are rare under expansion conditions, occasionally these are generated providing a visual way to clonally expand correctly targeted organoids.

### Quantitative PCR Analysis.

Organoid RNA was isolated using a RNAeasy kit (QIAGEN), following the manufacturer’s protocol. qPCR analysis was performed using biological and technical duplicates as described before (35). qPCR primers used are described in *SI Appendix,* Table S2.

### Organoid Lysis for Intracellular Peptide Hormone Extraction.

To extract intracellular peptide hormones from organoids, we first lysed the organoids in 8 M Urea in 50 mM ammonium bicarbonate (pH 8.5). Organoids were homogenized by vortexing, followed by sitting at 4 °C for 30 min. The lysates were snap frozen and stored at −80 °C until peptide extraction.

### Peptide Hormone Extraction.

For intracellular peptide hormone extraction, knock-out and wild-type organoid clones’ lysates were slowly thawed at 4 °C and spun down for 10 min at 20,000 × g at 4 °C, and the supernatants, containing proteins and peptides, were kept. Peptides were extracted by the methanol–chloroform method, as follows: 1 volume of sample was sequentially and thoroughly mixed with 4 volumes of methanol (Sigma-Aldrich), 1 volume of chloroform (Sigma-Aldrich), and 3 volumes of water. The mixture was centrifuged at 5,000 rpm for 10 min at 4 °C and the upper layer, containing the water-soluble peptide fraction, was dried down in a vacuum centrifuge. Then, 3 volumes of methanol were incorporated and centrifuged at 5,000 rpm for 10 min at 4 °C. The organic fraction was kept, and liquid chromatrography–tandem mass spectrometry (LC-MS/MS) analysis confirmed that no peptides were present in this fraction.

The dried down peptide samples were resuspended in a reduction buffer (4 mM dithiothreitol [DTT] in 50 mM ammonium bicarbonate) and gently mixed for 1 h at RT. Following reduction, samples were alkylated in 16 mM iodoacetamide for 30 min at RT, and reactions were quenched by addition of 4 mM DTT. Peptide samples were acidified to 5% formic acid (FA) and loaded into an Oasis HLB μElution plate (Waters) by gently applying positive pressure. Wells were washed with 200 μL 0.1% FA in water followed by a low organic wash with 5% methanol in 1% FA in water. Peptides were eluted twice by addition of 30 μL of 60% methanol in 10% FA in water. Samples were dried down in a vacuum centrifuge and kept at −20 °C until LC-MS/MS injection.

For extracellular peptide hormone extraction, 1 mL of knock-out and wild-type organoid clone supernatants were slowly thawed at 4 °C, acidified to 5% FA, and loaded into an Oasis HLB μElution plate (Waters) to be processed as previously described. Extracted peptides were dried down in a vacuum centrifuge and resuspended in a reduction buffer (4 mM DTT in 50 mM ammonium bicarbonate) and gently mixed for 1 h at RT. Following reduction, samples were alkylated in 16 mM iodoacetamide for 30 min at RT, and reactions were quenched by addition of 4 mM DTT. Samples were acidified to 5% FA and stored at −20 °C until LC-MS/MS injection.

All the steps, unless indicated otherwise, were performed at 4 °C to avoid peptide degradation, and all samples were collected and stored in low-binding tubes (LoBind, Sigma Aldrich), to minimize peptide losses.

### LC-MS/MS Analysis of Intracellular and Extracellular Peptidomes.

For mass spectrometry analysis, spectral data were acquired with an Ultimate 3000 system (Thermo Fischer Scientific) coupled to an Orbitrap Exploris 480 mass spectrometer (Thermo Fischer Scientific). Peptides were trapped (Dr Maisch Reprosil C18, 3 µM, 2 cm × 100 µM) before being separated on an analytical column (Agilent Poroshell, EC-C18, 2.7 µM, 50 cm × 75 µM). Solvent B consisted of 80% acetonitrile in 0.1% FA. Trapping of peptides was performed for 2 min in 9% B followed by peptide separation in the analytical column using a gradient of 13 to 44% B in 95 min. After peptide separation, gradients were followed by a steep increase to 99% B in 3 min, a 5-min wash in 99% B, and a 10-min re-equilibration at 9% B. Flow rate was kept at 300 nL/min. The mass spectrometer was operated in data-dependent mode. Peptides were ionized in a nanospray electrospray ionization source at 2 kV and focused at 70% amplitude of the radiofrequency lens to improve the ionization of bigger species. Full-scan MS1 spectra from 375 to 1,600 *m/z* were acquired in the Orbitrap at a resolution of 60,000 with the automatic gain control (AGC) target set to 3 × 10^6^ and under automated calculation of maximum injection time. Cycle time for MS2 fragmentation scans was set to 1 s. Only peptides with charged states 2 to 10 were fragmented, and dynamic exclusion was set to a duration of 16 s. Fragmentation was done using stepped higher-energy collisional dissociation (HCD)-normalized collision energies of 24 and 28%. Fragment ions were accumulated until a target value of 1 × 10^5^ ions was reached or under a maximum injection time of 300 ms, with an isolation window of 1.4 *m/z* before injection in the Orbitrap for MS2 analysis at a resolution of 15,000.

To validate the presence of glucagon and obtain even better spectral fragmentation, we set up a PRM assay using the same liquid chromatography–mass spectrometry setup, solvents, and trapping of the peptides on the trap column as described above. Separation on the analytical column was performed in 65 min using a gradient of 20 to 50% B. After peptide separation, identical wash and equilibration were performed as previously described. Flow rate was kept at 300 nL/min. The mass spectrometer was operated in multiplexed mode, obtaining a MS1 scan followed by unscheduled PRM on the precursors detailed in the inclusion list (*SI Appendix,* Table S3). An optimized RF lens amplitude was set to 70%, and MS1 scans were acquired from 375 to 1,600 *m/z* at a resolution of 120,000, with the normalized AGC target set to 100% and injection time set to 300 ms. PRM scans were acquired at a resolution of 240,000, with a quadrupole isolation width of 0.4 *m/z*, the normalized AGC target set to 3,000%, and injection time set to 512 ms. A stepped HCD approach was utilized with normalized collision energies set to 27, 30, and 33%.

### Peptidomics Database Search.

Raw data were searched using the ultrafast search engine MSFragger (version 3.3) (36) through Proteome Discoverer software (version 2.5.0.400). Spectra were extracted, precursors with a mass between 350 and 12,000 Da were kept, and the signal-to-noise ratio was set to 1.5. To minimize search space, data were searched against a tailored database containing 369 reviewed *Homo sapiens* proteins (downloaded from Uniprot on October 2021) that contained any peptide annotation or a chain length of up to 60 amino acids and wereappended with the nonhormone identified proteins retrieved from a search using the SwissProt human database (840 proteins). For spectral searches, precursor mass tolerances were set to ±20 ppm and fragment mass tolerances to ±0.02 Da. The digestion enzyme was set to unspecific, with peptides ranging from 7 to 65 amino acids in length, a maximum charge state for theoretical fragments to match of 4, and automatic clipping of N-terminal methionine. Carbamidomethylation of cysteines was set as static modification, while methionine oxidation, N-terminal acetylation, and C-terminal amidation were set as variable modifications, with a maximum co-occurrence of 3 variable modifications per peptide. False discovery rate (FDR) calculations were done using Philosopher (Peptide Prophet, version 4.0) (37), and only high-confidence peptides (1% FDR) were kept. Minora Feature Detector and Feature Mapper nodes were used for untargeted MS1-based peptide quantification, with default parameters except for the maximum retention time shift and the mass tolerance, which were set to 5 min and 5 ppm, respectively, in the chromatographic alignment window. Total peptide and protein abundance were normalized across all files, and normalized abundance was used for downstream analyses.

### Peptidomics Data Analysis.

For PCSK-like hormone processing analysis, protein or peptide abundance is log2 transformed, z-scaled, and plotted across replicates. For glucagon, bioactive regions annotated in Uniprot were extracted from our data and plotted separately. To assess statistical significance on relevant comparisons, two-sided Student’s *t* test *P* values were computed on raw, log2-transformed, protein or peptide abundance for the prohormones, and peptides are displayed in Fig. 3 *A* and *B* (*SI Appendix*, Fig. S7). Amidated versions of GRPP and oxyntomodulin, where the amidation site was not followed by a glycine residue, were not included in the plots. Glucagon peptide validation was performed by manual inspection of the MS1 traces on each file using Skyline Daily (version 21.2.1.377) (38). Isotopic distributions that highly resembled the theoretical isotope distribution of the ion (idotp ≥ 0.88), with a similar peak shape, low mass error, and eluting at expected retention time range (min 89 to 91) were kept. Total abundance was calculated by summation of intensities for each isotope (up to M+4) in each sample.

PRM data were analyzed using Byonic (version 4.3.4) and Skyline Daily. Byonic searches were set to ±10 ppm precursor tolerance, set to ±20 ppm fragment mass tolerance, and limited to peptides with 0 miscleavages. A maximum of 2 variable modifications was set including methionine oxidation and C-terminal amidation and using a fixed cysteine carbamidomethylation modification. Spectra were searched against a database of 8 glucagon peptides and decoy peptides. Only high-confidence peptides were considered (1% FDR). The identified glucagon peak was further visually assessed using Skyline. Important aspects were similar peak shape of all transitions and low measured MS1 and MS2 mass errors and MS1 isotope distribution. Spectra were plotted using Interactive Peptide Spectral Annotator (39) and refined in Illustrator.

For CPB1, CPE, and DPP4 specificity analysis, peptides found in all injection replicates of at least one organoid clone were included for statistical analysis. Peptide abundance was log2 transformed, missing values were imputed according to the normal distribution of each sample, and Student’s *t* test was used to determine which peptides were significantly changing in response to enzyme knockouts when compared to wild types. Peptides were considered significantly changing if corrected *P* values (*q*-value, calculated using the permutation method with 250 iterations) were inferior to 0.05. Peptide abundance was scaled (z-score) and hierarchically clustered to reveal the subset of peptides accumulating in response to enzyme knockout. The N-terminal or C-terminal cleavage specificities were plotted using the R package ggseqlogo (40). Data processing was done in Perseus (version 1.6.14.0) (41), while data visualization was done using basic R packages and refined in Illustrator.

### Statistical Analysis.

At all instances, unpaired Student’s *t* tests were performed (*P < 0.05; **P < 0.01; ***P < 0.001). Exact values of n and measures are described in legends of each figure. No statistical methods were used to predetermine sample size. In *SI Appendix*, Fig. S1*G*, we could not detect PCSK2 in samples treated without BMP after 40 amplification cycles; for statistical analysis, expression at cycle threshold 40 was assumed for PCSK2 minus BMP.

For quantification in Fig. 3*H*, at least 25 Glucagon- or GLP-1-positive cells were counted in crypts and in villi, in sections from 5 separate donors.

## Supplementary Material

Supplementary File

Supplementary File

Supplementary File

Supplementary File

## Data Availability

Peptidomics data, together with curated results, have been deposited to ProteomeXchange Consortium via the PRIDE repository (42) and can be accessed through the identifier PXD033369 (43). The bulk RNA-sequencing data generated in this study has been deposited in the Gene Expression Omnibus (GEO) under accession code GSE212636 (44).
