# Supplementary material for: Mapping prohormone processing by proteases in human enteroendocrine cells using genetically engineered organoid models
Source: Proc Natl Acad Sci U S A. 2022 Nov 7;119(46):e2212057119. doi: 10.1073/pnas.2212057119 (PMC9674236; doi:10.1073/pnas.2212057119)
Supplement: Supplementary File [file pnas.2212057119.sapp.pdf]

PNAS

Supplementary Information for

Mapping the role of proteases in prohormone processing in human enteroendocrine cells using genetically engineered organoid models

Joep Beumer, Julia Bauzá-Martinez, Tim S. Veth, Veerle Geurts, Charelle Boot, Hannah Gilliam-Vigh, Steen S. Poulsen, Filip Knop, Wei Wu, Hans Clevers

Wei Wu and Hans Clevers
Email: h.clevers@hubrecht.eu

This PDF file includes:

Figures S1 to S8
Table 1 to 3
Legends for Datasets S1 to S3
SI References

Other supplementary materials for this manuscript include the following:

Datasets S1 to S3

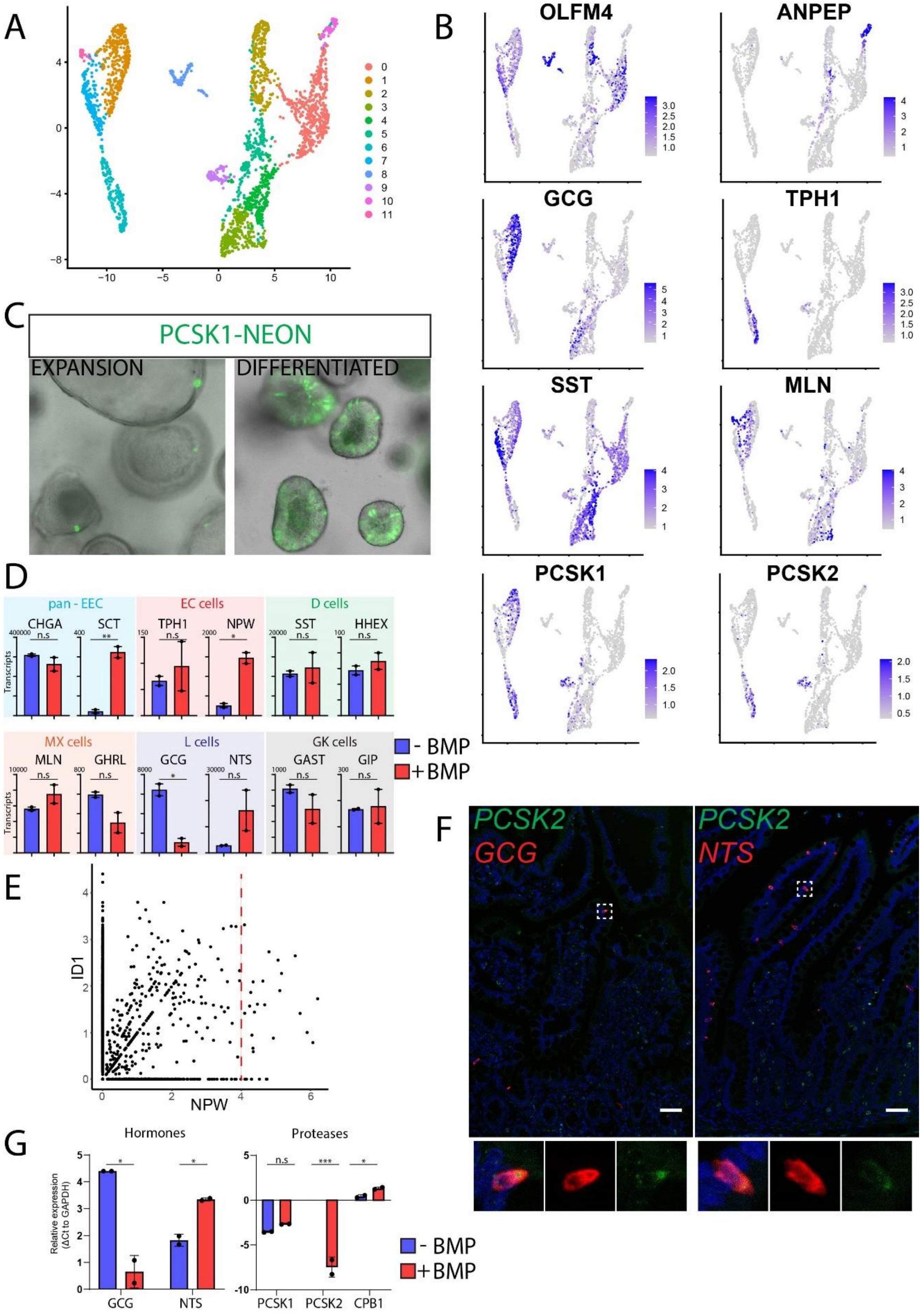

A
B
OLFM4
ANPEP
GCG
TPH1
SST
MLN
PCSK1
PCSK2
C
PCSK1-NEON
EXPANSION
DIFFERENTIATED
D
pan - EEC
EC cells
D cells
MX cells
L cells
GK cells
CHGA
SCT
TPH1
NPW
SST
HHEX
MLN
GHRL
GCG
NTS
GAST
GIP
Transcripts
- BMP
+BMP
E
ID1
NPW
F
PCSK2
GCG
PCSK2
NTS
G
Hormones
Proteases
Relative expression (ΔCt to GAPDH)
GCG
NTS
PCSK1
PCSK2
CPB1
- BMP
+BMP

Fig. S1. Expression and BMP-dependency of EEC hormones and proteases

(A) UMAP showing clustering of different EEC populations derived from organoid cultures based on previously performed single-cell RNA-sequencing (1).

(B) Feature map showing normalized transcript levels of selected markers on dataset from (A).

(C) Overlay of brightfield and fluorescent image from PCSK1^{mNeon} organoids in culture. PCSK1-neon positive cells are strongly increased upon differentiation.

(D) Graphs depicting hormone expression revealed by bulk RNA-sequencing of EEC-differentiated organoids in the presence and absence of BMPs.

(E) NPW and ID1 expression plotted from a previously published single cell RNA-sequencing atlas of the gut epithelium (2). Cells expressing the highest levels of NPW (an artificial cut-off of 4 transcripts indicated by red line) are ID1-positive.

(F) Fluorescent in situ hybridization (RNAscope) showing low expression of *PCSK2* in *GCG-* and *NTS*-expressing cells in the human small intestinal epithelium.

(G) qPCR analysis on sorted NTS-producing cells in the presence and absence of BMP. BMP stimulates the production of PCSK2. Expression was undetectable in absence of BMP (N.D: non-detectable). Experiment was performed n=2 independent experiments, and Ct values are average of technical replicate. Data is depicted as ΔCt compared to GAPDH.

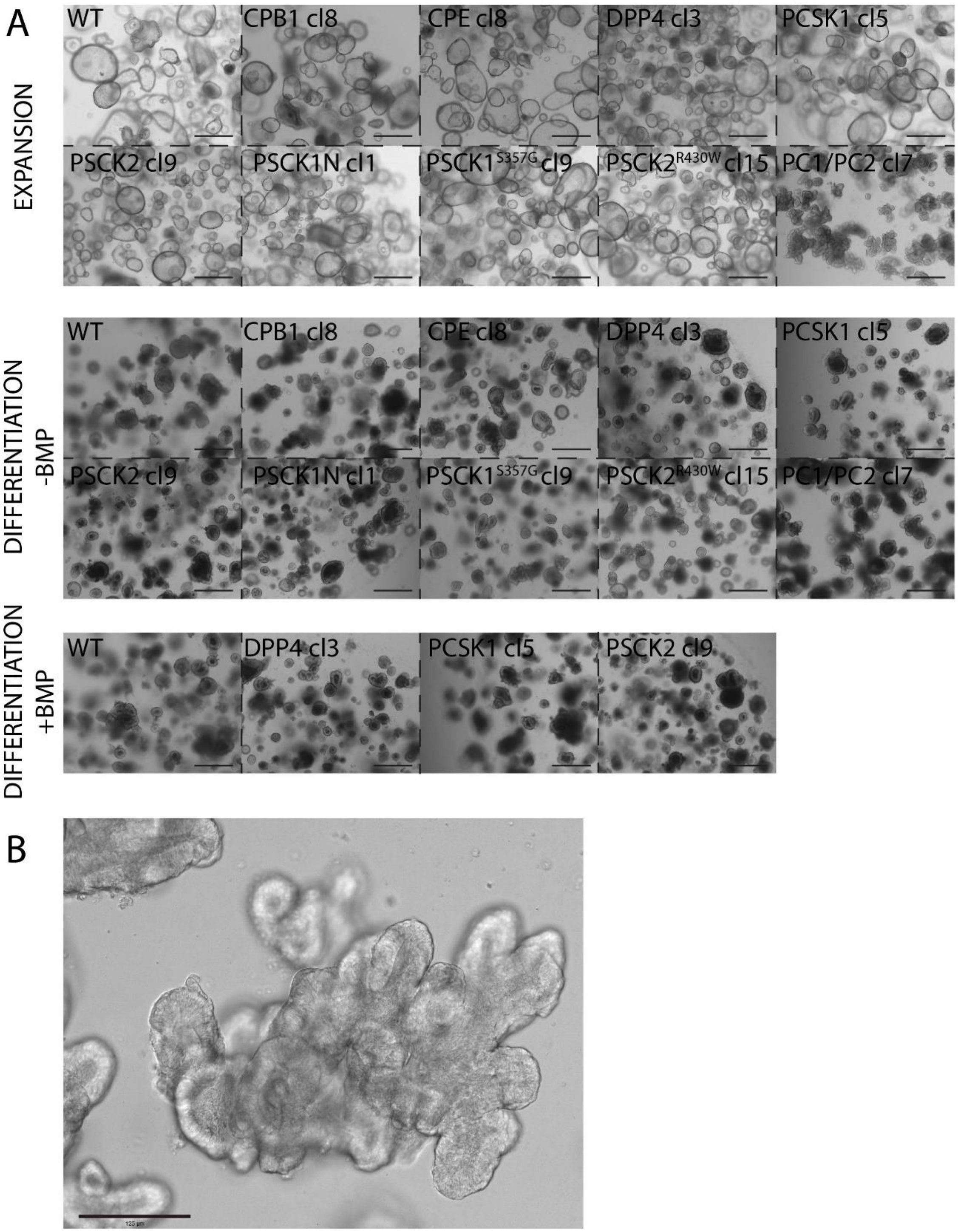

Fig. S2. Biobank of protease mutant organoids

(A) Brightfield images of expanding and differentiated organoids harboring different protease mutations. Scale bars are 500 µm.

(B) Zoom-in brightfield image of PCSK1/PCSK2 double mutant organoids. Scale bar is 125 µm.

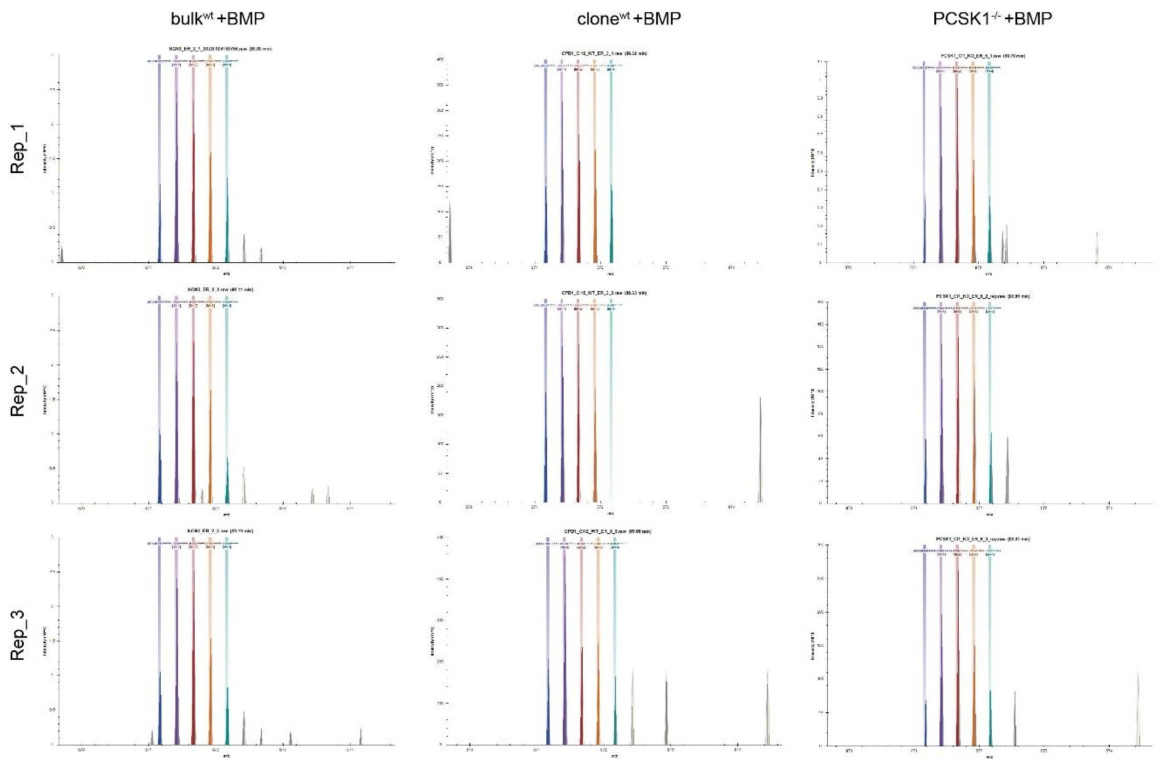

Fig. S3. Extracted MS1 isotopic distributions for glucagon peptide in BMP-treated samples

MS1 isotopic distributions are extracted from raw files based on manual inspection using Skyline. The precursor ion (m/z = 871.1612, z = 4) as well as isotopes M+1 to M+4 are plotted. Retention time, similarity to the theoretical ion isotopic distribution (idotp) and low mass error are taken into consideration to determine the ion’s presence or absence.

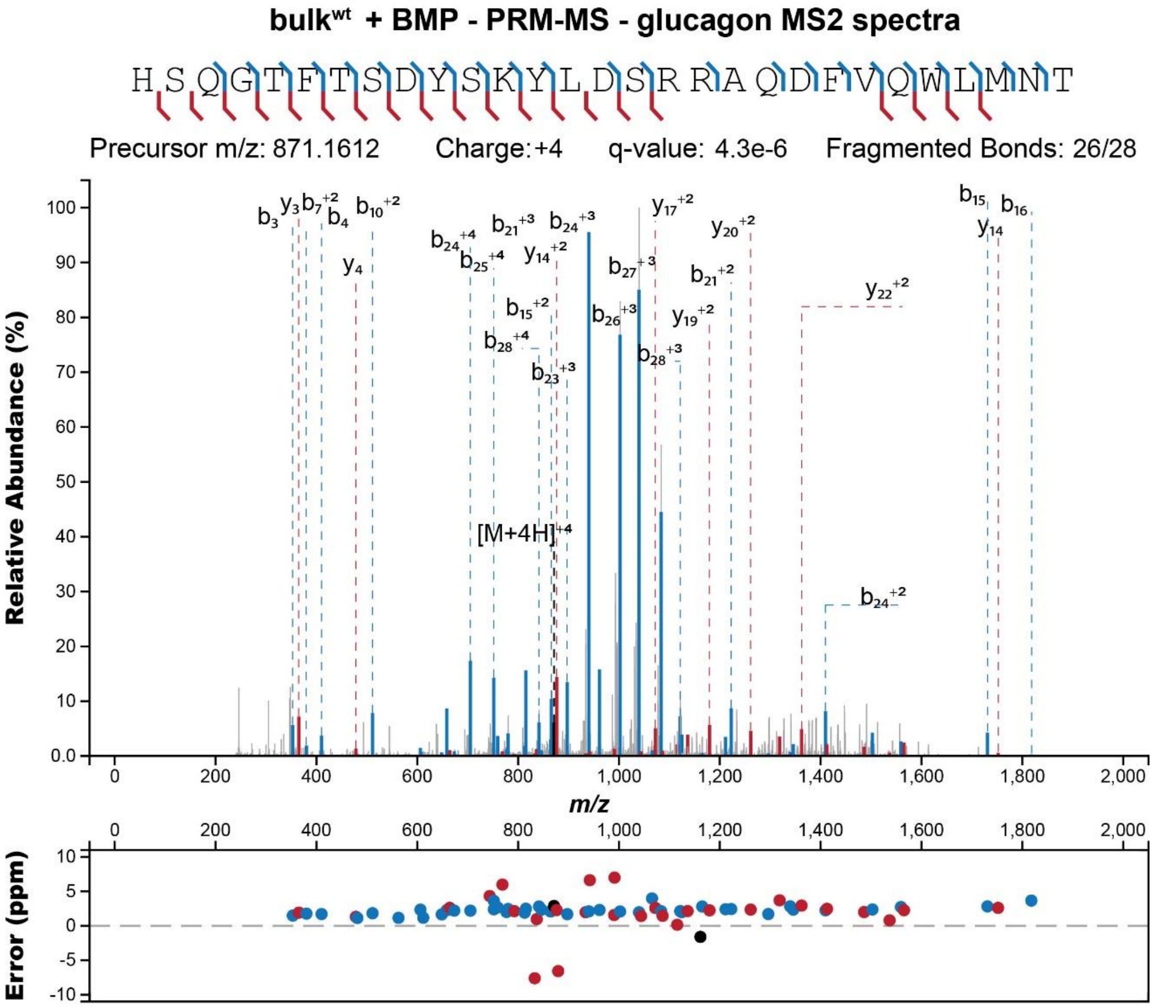

Fig. S4. Annotated MS2 spectra for glucagon measured by PRM-MS

Measured MS2 spectrum of the glucagon peptide acquired at a 240k resolution. Only the precursor peak, b-ions and y-ions are used to visualize the spectrum using a 10ppm cut-off. The spectrum shows almost complete fragment annotation of the glucagon peptide with 26 out of 28 fragmented bonds found. Only the highest intensity peaks are labeled in the MS2 spectrum. Spectrum quality was visually assessed and statistically validated using a q-value calculated using a target-decoy strategy, which confirmed glucagon peptide identification.

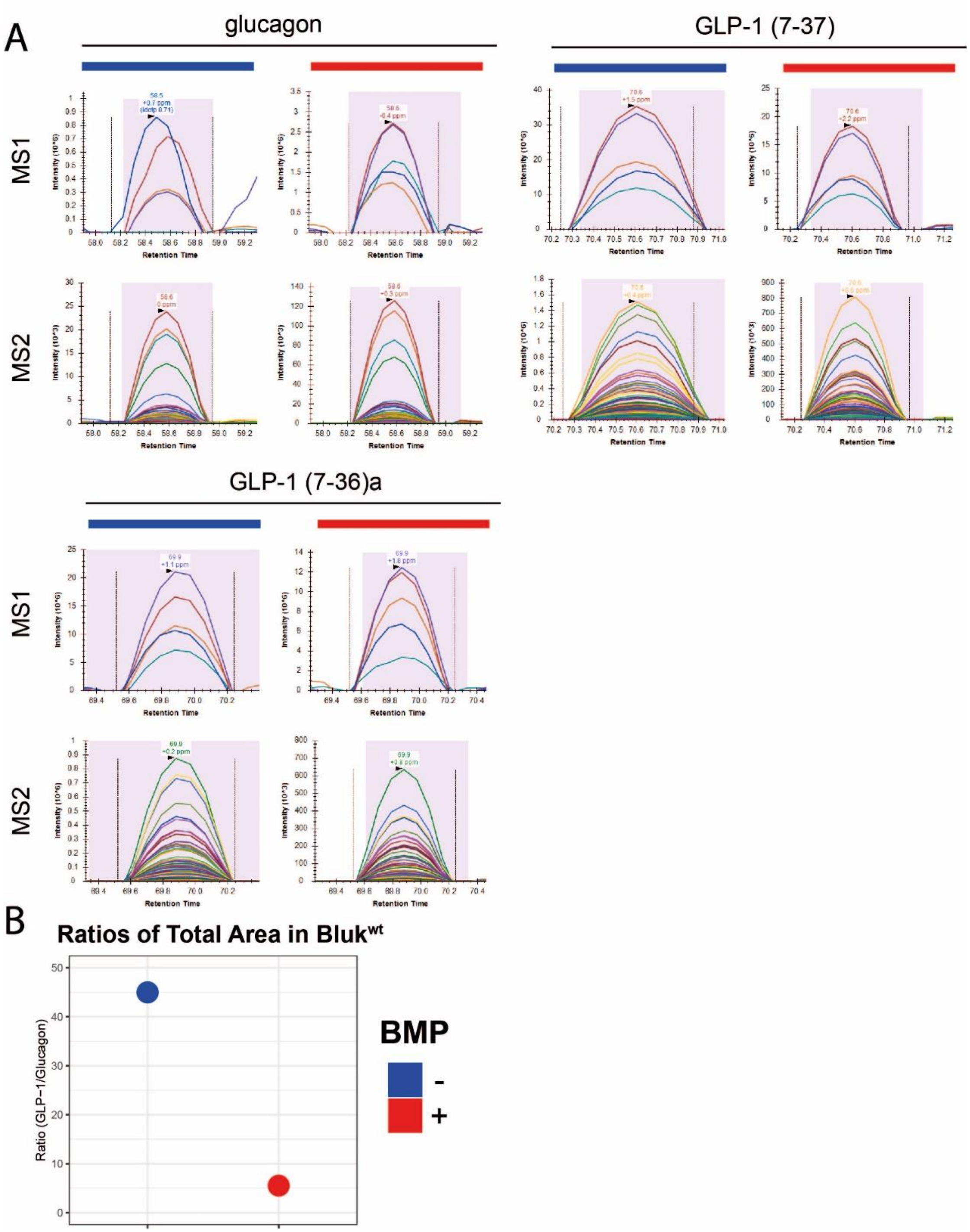

Fig. S5. LC-MS/MS traces of glucagon and active GLP-1 peptides as measured by PRM-MS in BMP-treated and -untreated bulk wildtype organoids

(A) MS1 and MS2 traces for bioactive glucagon, GLP-1 (7-37) and amidated GLP-1 (7-36) as measured by PRM-MS in BMP-treated and BMP-untreated bulk wildtype organoids. The precursor ion (m/z = 871.1612, z = 4) as well as isotopes M+1 to M+4 are displayed for MS1, and

all matching transitions are displayed for MS2. Mass error, in parts per million (ppm), and retention time are shown on top of the peaks.

(B) The GLP-1:glucagon ratio of peak area is displayed for BMP-treated and BMP-untreated bulk wildtype organoids. The total peak area for GLP-1 is calculated by averaging GLP-1 (7-37) and GLP-1 (7-36)a areas.

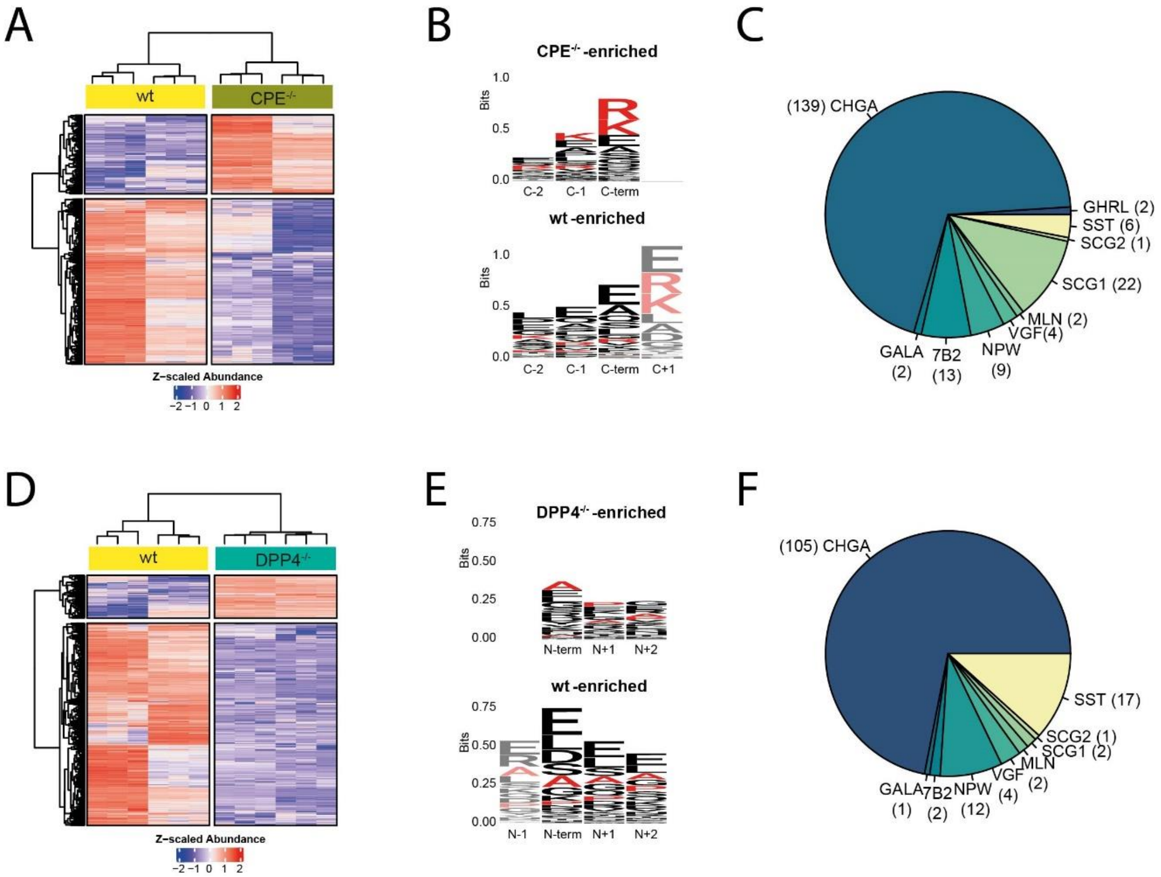

Fig. S6. Extracellular substrate specificity of EEC exopeptidases

(A) Significantly changing hormone-derived peptides in response to CPE knockout (CPE-/-) when compared to wildtype organoids. Bulk and a clonal wildtype line and two CPE-/- clones were included. Three technical replicates were measured for all samples.

(B) Sequence logo of the C-termini of peptides accumulating in response to CPE knockout (CPE-/- -enriched) extracellularly, and peptides accumulating in wildtype (wt-enriched) extracellularly. Lysine (K) and arginine (R) are highlighted in red.

(C) Pie chart indicating the substrate repertoire of peptides accumulating in CPE knockouts extracellularly.

(D) Significantly changing hormone-derived peptides in response to DPP4 knockout (DPP4-/-) when compared to wildtype organoids. Bulk and a clonal wildtype line and two DPP4-/- clones were included. Three technical replicates were measured for all samples.

(E) Sequence logo of the C-termini of peptides accumulating in response to DPP4 knockout (DPP4-/--enriched) extracellularly, and peptides accumulating in wildtype (wt-enriched) extracellularly. Lysine (K) and arginine (R) are highlighted in red.

(F) Pie chart indicating the substrate repertoire of peptides accumulating in DPP4 knockouts extracellularly.

A

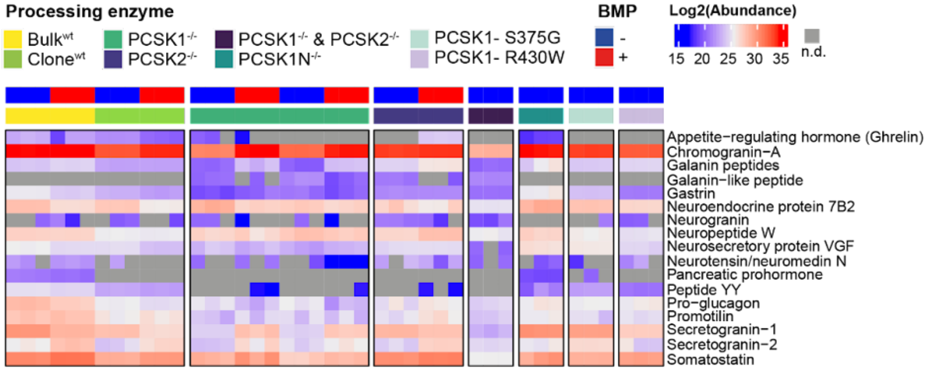

Student's T-test p-value

wt(-) vs. PCSK1(-)	wt(-) vs. PCSK2(-)	wt(-) vs. PCSK1/2(-)	wt(-) vs. PCSK1N(-)	wt(-) vs. PCSK1mut S375G(-)	wt(-) vs. PCSK2mut R430W(-)	wt(+) vs. PCSK1(+)	wt(+) vs. PCSK2(+)	wt(-) vs. wt(+)	PCSK1(-) vs. PCSK1(+)	PCSK2(-) vs. PCSK2(+)	
0.003	-	-	0.000	-	-	-	0.006	0.032	-	-	**Appetite-regulating hormone (Ghrelin)**
0.001	0.849	0.000	0.232	0.803	0.098	0.993	0.034	0.090	0.000	0.005	**Chromogranin-A**
0.000	0.034	0.000	0.018	0.108	0.024	0.809	0.005	0.650	0.000	0.000	**Galanin peptides**
-	-	-	-	-	-	-	-	-	0.026	-	**Galanin-like peptide**
0.000	0.008	0.001	0.107	0.522	0.003	0.000	0.027	0.176	0.925	0.523	**Gastrin**
0.402	0.024	0.000	0.001	0.883	0.000	0.002	0.736	0.000	0.008	0.305	**Neuroendocrine protein 7B2**
0.098	0.015	0.640	-	-	0.578	0.034	0.991	0.553	0.000	0.165	**Neurogranin**
0.035	0.349	0.146	0.224	0.351	0.046	0.062	0.012	0.796	0.405	0.000	**Neuropeptide W**
0.000	0.080	0.000	0.002	0.014	0.023	0.007	0.113	0.006	0.834	0.285	**Neurosecretory protein VGF**
0.020	0.201	0.011	0.010	-	0.867	0.037	0.763	0.705	0.369	0.440	**Neurotensin/neuromedin N**
-	-	-	0.000	-	-	-	-	0.015	-	-	**Pancreatic prohormone**
-	-	-	0.010	0.080	0.003	0.000	0.000	0.573	-	-	**Peptide YY**
0.025	0.119	0.036	0.377	0.203	0.282	0.001	0.881	0.361	0.000	0.228	**Pro-glucagon**
0.001	0.033	0.005	0.118	0.019	0.029	0.000	0.232	0.569	0.850	0.030	**Promotilin**
0.000	0.458	0.000	0.123	0.451	0.039	0.942	0.750	0.050	0.000	0.000	**Secretogranin-1**
0.097	0.297	0.524	0.504	0.019	0.622	0.012	0.581	0.000	0.000	0.000	**Secretogranin-2**
0.006	0.373	0.000	0.815	0.132	0.704	0.014	0.619	0.118	0.055	0.001	**Somatostatin**

B

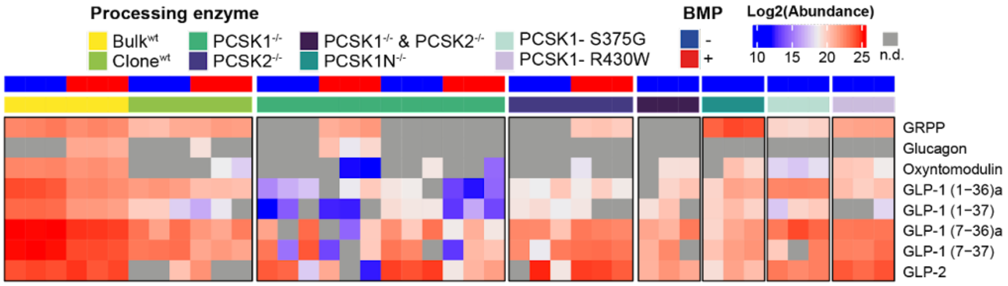

e Student's T-test p-valu

wt(-) vs. PCSK1(-)	wt(-) vs. PCSK2(-)	wt(-) vs. PCSK1/2(-)	wt(-) vs. PCSK1N(-)	wt(-) vs. PCSK1mut S375G(-)	wt(-) vs. PCSK2mut R430W(-)	wt(+) vs. PCSK1(+)	wt(+) vs. PCSK2(+)	wt(-) vs. wt(+)	PCSK1(-) vs. PCSK1(+)	PCSK2(-) vs. PCSK2(+)	
-	-	-	0.010	0.014	0.730	0.177	0.001	0.626	-	-	**GRPP**
-	-	-	-	-	-	0.163	-	-	-	-	**Glucagon**
-	-	0.000	0.005	0.000	0.003	0.002	-	0.167	-	-	**Oxyntomodulin**
0.000	0.003	0.021	0.038	0.602	0.011	0.003	0.011	0.055	0.320	0.913	**GLP-1 (1-36)a**
0.028	0.167	0.445	0.416	0.197	-	0.001	-	0.423	0.113	-	**GLP-1**
0.060	0.071	0.066	0.024	0.694	0.341	0.004	0.505	0.488	0.030	0.249	**GLP-1(7-36)a**
0.123	0.037	0.041	0.009	0.043	0.120	0.011	0.749	0.124	0.141	0.232	**GLP-1(7-37)**
0.888	0.616	0.175	0.076	0.644	0.663	0.050	0.540	0.659	0.072	0.387	**GLP-2**

Fig. S7. Raw abundance profiles and statistical significance of prohormones and their derived peptides.

(A) Related to Figure 3A. Raw, log2 transformed, LC-MS/MS abundance profiles of the diverse prohormones measured in the intracellular peptidome of wildtype (wt) and mutant organoids. Concentration of measured peptides was higher in the parental, non-clonal organoid ('Bulk') compared to clonal lines due to higher differentiation efficiency. Samples where the prohormone was not detected (n.d.) are displayed in gray. BMP-untreated (-) is shown in blue and BMP-treated (+) is shown in red. The Student's T-test p-values for relevant comparisons are displayed on the table underneath for each prohormone. For comparisons with less than 2 measurements, p-values are not calculated and a "-" is shown.

(B) Related to Figure 3B. Raw, log2 transformed, LC-MS/MS abundance profiles of the bioactive peptides derived from proglucagon. For amidated peptides, an "a" is annotated after the peptide name. Samples where the peptide was not detected (n.d.) are displayed in gray. BMP-untreated (-) is shown in blue and BMP-treated (+) is shown in red. The Student's T-test p-values for relevant comparisons are displayed on the table underneath for each peptide. For comparisons with less than 2 measurements, p-values are not calculated and a "-" is shown.

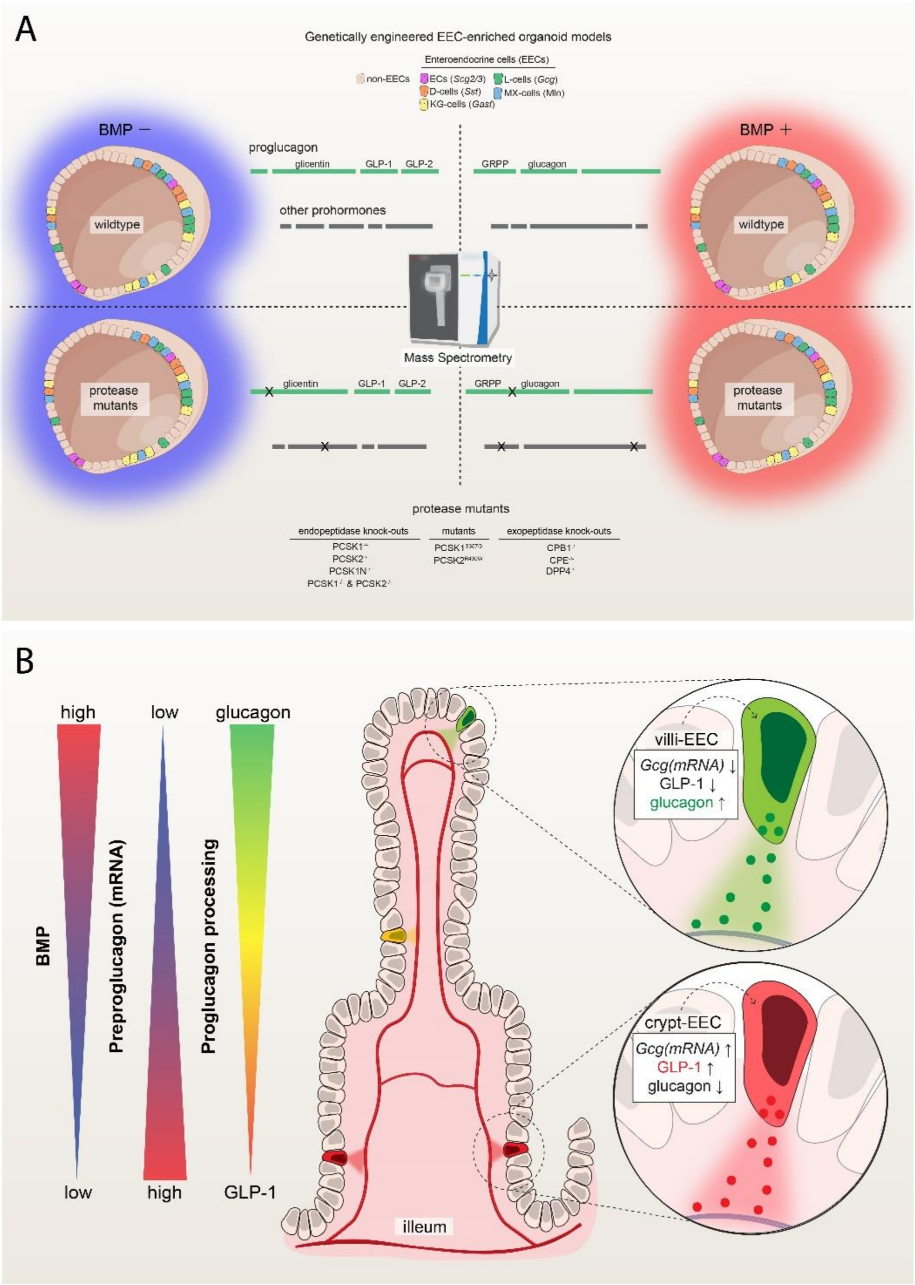

Fig. S8. Genetically engineered organoids to model prohormone processing

(A) Overview of genetically engineered protease mutants generated in this study. Organoids were cultured with or without BMP. Intracellular peptides were collected and analyzed by mass spectrometry. Comparison of measured peptides across conditions allows for precise mapping of BMP- and protease targets simultaneously over multiple prohormones.
(B) Model on proglucagon processing in the intestinal epithelium. L-cells downregulate Preproglucagon messenger along the crypt-villus axis, under influence of non-epithelial signals including BMPs. Additionally, BMP can stimulate increased processing of Proglucagon prohormone to glucagon instead of GLP-1.

Table 1 Overview of protease mutant organoids
All clones included are homozygous frameshift mutations, or homozygous base edits without off-target. For PCSK2-R430W, only one correctly edited heterozygous clone was generated.

Gene	Clone number	Allele 1	Allele 2
CPB1	8	Min13	Min13
	11	Plus 1	Plus 5
	15	Min1	Min16
CPE	4	Min17	Min17
	8	Min8	Min16
	11	Min1	Min1
	12	Plus 1	Min 1
DPP4	1	Plus 1	Plus 1
	2	Plus 1	Plus 1
	3	Plus 1	Plus 1
	4	Plus 1	Plus 1
	5	Plus 1	Min2
	6	Plus 1	Plus 1
	7	Plus 1	Plus 1
	8	Plus 1	Plus 1
	9	Plus 1	Plus 1
	10	Plus 1	Plus 1
	11	Plus 1	Plus 1
	12	Plus 1	Plus 1
PCSK1N	1	Min 51	x
	2	Plus 1	x
	5	Plus 1	x
PCSK1	1	Min 2	Min 2
	3	Min 23	Min 23
	5	Min 14	Min 14
	7	Plus 13	Min 14
PCSK2	1	Plus 1	Plus 2
	9	Plus 1	Plus 1
	11	Plus 1	Min 23
	15	Plus 1	Plus 1
PCSK2 (in PCSK1 clone 1)	2	Plus 1	Min 17
	7	Min 1	Plus 1
	11	Plus 1	Min 16
PCSK1 S357G	3	S357G	S357G
	9	S357G	S357G
PCSK2 R430W	15	R430W	WT

Table 2 Oligos used in this study

Genotyping primers

*Indicates if PCR primer was used as sequencing primer

PCSK1KO_FW_PCR*	ACATCCGCCTGTGGTTTAGAG
PCSK1KO_REV_PCR	CAAAAGGCGTTGTAGCTGAGTG
PCSK2KO_FW_PCR	cagggagggtttcctgCAAT
PCSK2KO_REV_PCR*	GGCCATCATGCCCAGTATCT
PCSK1NKO_FW_PCR	ACGGACGCTGACAAACTCTT
PCSK1NKO_REV_PCR	GGTAAGCAGGCTTCCCAGTT
PCSK1NKO_SEQ	TTCCCCCACCTCATTTTCCC
CBP1KO_FW_PCR	GAGGTTTGGGCTGTGGAAGA
CPB1KO_REV_PCR	GGTAAATAGCGCGTCCCTCA
CPB1KO_SEQ	CTCTGGAGCAGCTGAAGGTC
CPEKO_FW_PCR	CTGACACTCATTCAGCCGGG
CPEKO_REV_PCR	ccaccagtcccaccctct
CPEKO_SEQ	CCTTACCAGGCTCATGGACG
PCSK1_S357G_FW_PCR	CACACATGTAGTCGTTGGGGA
PCSK1_S357G_REV_PCR	TTAGGGGAGACAGGGGAAGG
PCSK1_S357G_SEQ	TGCCAGCCTTTCAAGTGAGT
PCSK2_R430W_FW_PCR	TACAAGAGGTCTGTGTCCTGAGTAA
PCSK2_R430W_REV_PCR	CAGCAGAGGGACCACTACCC
PCSK2_R430W_SEQ	TCACCCTGCCTTTTGCCC

qPCR primers

GCG	Forward	ACATTGCCAAACGTCACGATG
GCG	Reverse	TCTGCGGCCAAGTTCTTCAA
NTS	Forward	TGCTTTAGATGGCTTTAGCTTGG
NTS	Reverse	TTCCTGGATTAACTCCCAGTGT
PCSK1	Forward	CTGGATGGCATTGTGACGGAT
PCSK1	Reverse	GCCCCAGCTTGCACTGTAAA
PCSK2	Forward	GGGAAAGGTGTTACCATTGGAA
PCSK2	Reverse	CCAGTCATCTGTGTACCGAGG
CPB1	Forward	CCAGCACGACCCAGATTGAC
CPB1	Reverse	AACCCGGCTATCAAACTGAGC
GAPDH	Forward	GGAGCGAGATCCCTCCAAAAT
GAPDH	Reverse	GGCTGTTGTCATACTTCTCATGG

Table 3 PRM inclusion list

Name	Compound	m/z	z
Oxyntomodulin	HSQGTFTSDYSKYLDSRRAQDFVQWLMNTKRNRNNIA (light)	742.2011	6
GLP-1(1-36a)	HDEFERHAEGTFTSDVSSYLEGQAAKEFIAWLVKGR[-0.984016] (light)	822.8079	5
GLP-1(7-36a)	HAEGTFTSDVSSYLEGQAAKEFIAWLVKGR[-0.984016] (light)	824.9229	4
GLP-1(1-37)	HDEFERHAEGTFTSDVSSYLEGQAAKEFIAWLVKGRG (light)	834.409	5
GLP-1(7-37)	HAEGTFTSDVSSYLEGQAAKEFIAWLVKGRG (light)	839.4243	4
GRPP	RSLQDTEEKSRSFSASQADPLSDPDQMNED (light)	846.6287	4
Glucagon	HSQGTFTSDYSKYLDSRRAQDFVQWLMNT (light)	871.1612	4
GLP-2	HADGSFSDEMNTILDNLAARDFINWLIQTKITD (light)	941.9611	4

Dataset S1 Normalized transcript count of control and BMP-treated, EEC-differentiated organoids.

Dataset S2 Differentially expressed genes between BMP-treated and -untreated EEC-differentiated organoids.

Dataset S3 Hormone-derived peptides accumulating or decreasing in CPE-/-, CPB1-/- and DPP4-/- organoids when compared to wildtypes

SI References

1. J. Beumer, *et al.*, High-Resolution mRNA and Secretome Atlas of Human Enteroendocrine Cells. *Cell* **181**, 1291-1306.e19 (2020).

2. R. Elmentaite, *et al.*, Cells of the human intestinal tract mapped across space and time. *Nature* **597**, 250–255 (2021).
